# Decolourisation of Different Dyes by two *Pseudomonas* Strains Under Various Growth Conditions

**DOI:** 10.1007/s11270-013-1846-0

**Published:** 2014-01-22

**Authors:** Ewa Zabłocka-Godlewska, Wioletta Przystaś, Elżbieta Grabińska-Sota

**Affiliations:** Environmental Biotechnology Department, Silesian University of Technology, Akademicka 2A, 44-100 Gliwice, Poland

**Keywords:** *Pseudomonas*, Azo dye, Triphenylmethane dye, Decolourisation, Zootoxicity, Phytotoxicity

## Abstract

The aim of the present study was the decolourisation of mixture of two dyes belonging to different groups by two *Pseudomonas fluorescens* strains (Sz6 and SDz3). Influence of different incubation conditions on decolourisation effectiveness was evaluated. Dyes used in the experiment were diazo Evans blue (EB) and triphenylmethane brilliant green (BG). Another goal of the experiment was the estimation of toxicity of process by-products. Incubation conditions had a significant influence on the rate of decolourisation. The best results were reached in shaken and semistatic samples (exception Evans blue). After 24 h of experiment in semistatic conditions, BG removal reached up to 95.4 %, EB 72.8 % and dyes mixture 88.9 %. After 120 h, all tested dyes were completely removed. In most cases, dyes were removed faster and better by strain Sz6 than SDz3. At the end of the experiment, in majority of the samples, decrease of phyto- and zootoxicity was observed.

## Introduction

Synthetic dyes especially azo and triphenylmethane ones are used in many industrial branches for dyeing of wool, cotton, nylon, leather, paper, cosmetics, pharmaceuticals, food, plastic, petroleum products, etc. (Hamid and Rehman 2009; Koyani et al. 2013; Padamavathy et al. 2003; Somasiri et al. 2006; Swamy and Ramsay 1999; Tony et al. 2009; Younes et al. 2012). There are 100,000 commercially available dyes. Annual global dyes production reaches almost 7 × 10^7^ t. Among the 12 classes of dyes, the majority (70 %) are azo dyes containing one or more azo groups. Because of the imperfection of dyeing process, approximately 10–15 % of the synthetic dyes is released into the industrial waste, causing serious environmental problems worldwide (Hamid and Rehman 2009; Koyani et al. 2013; Tony et al. 2009; Silveira et al. 2009). Inadequate treatment of coloured wastewaters may lead to contamination of surface waters. The consequence is reduction of the penetration of light, photosynthetic activity and deficiency of oxygen. Synthetic origin of dyes and complex aromatic molecular structure cause that many ofthem are toxic and mutagenic, resistant to biological degradation and may be accumulated in the food chain (Azmi et al. 1998; Banat et al. 1996; Forgacs et al. 2004; Hamid and Rehman 2009; Koyani et al. 2013; Pointing and Vrijmoed 2000; Sani and Banerjee 1999). Increasing use of dyes causes an increase of their concentration in the environment and requires the development of new, environmentally and economically acceptable treatment technology. Currently available physicochemical methods (chemical oxidation, reverse osmosis, adsorption, flotation, precipitation, coagulation) are very effective but are costly, energy intensive, have limited use and can lead to the production of toxic by-products and a large amount of sludges (Eichlerova et al. 2006; Hamid and Rehman 2009; Hu 2001; Koyani et al. 2013; Tony et al. 2009). In comparison with the methods mentioned above, the methods based on microbial removal of dyes tend to be more attractive and are becoming more and more popular. Microbial degradation of dyes is gaining importance as an ecological, economical, less invasive alternative treatment techniques (Forgacs et al. 2004; Hamid and Rehman 2009; Koyani et al. 2013; Saratale et al. 2011; Silveira et al. 2009; Singh and Pakshirajan 2010; Srinivasan and Viraraghavan 2010; Tony et al. 2009; Younes et al. 2012; Zabłocka-Godlewska et al. 2012). Literature describes the participation of different taxonomic groups of organisms (bacteria, fungi, algae, plants) in the dyes decolourisation. The most popular are the technologies based on biodegradation/biotransformation of dyes by living bacteria or fungi. A lot of studies especially in case of fungi are concentrated on physical sorption on living or dead biomass. These technologies are more attractive than physical and chemical treatment methods due to low operating costs, high effectiveness and environmental harmlessness (An et al. 2002; Chen et al. 2003; Wang et al. 2012; Wu et al. 2009, 2012). Bacteria used in these technologies are applied as a single strain, co-cultures of strains or mixed bacterial cultures. The most popular genera of bacteria described in literature are *Pseudomonas*, *Bacillus*, *Sphingomonas*, *Aeromonas*, *Citrobacter*, *Escherichia*, *Desulphovibrio*, *Proteus*, *Schewanella* and *Alcaligenes* (An et al. 2002; Nigam et al. 1996; Saratale et al. 2011; Sharma et al. 2004; Tony et al. 2009; Wang et al. 2012).

It was proven that the efficiency of dye removal depends on the type of microorganisms and the process conditions. The most important factors are temperature, oxygen level, pH, additional available carbon and nitrogen sources, as well as the concentration of the dye and its chemical structure (Azmi et al. 1998; Banat et al. 1996; Forgacs et al. 2004; Hamid and Rehman 2009; Koyani et al. 2013; Padamavathy et al. 2003; Pointing and Vrijmoed 2000; Przystaś et al. 2009; Sani and Banerjee 1999; Saratale et al. 2011; Stolz 2001; Swamy and Ramsay 1999; Tony et al. 2009; Younes et al. 2012; Zabłocka-Godlewska et al. 2009).

Abilities of two strains of *Pseudomonas fluorescens* isolated from two different wastewater treatment plants for decolourisation of various structurally dyes were studied previously (Zabłocka-Godlewska et al. 2012). Five different dyes belonging to three different groups were examinated (two fluorone dyes, triphenylmethane brilliant green and crystal violet and azo Evans blue). Results of test of decolourisation potential showed significant differences between abilities of both tested strains. Triphenylmethane and azo dyes were more efficiently removed by Sz6 strain isolated from small treatment plant. Removal of triphenylmethane dyes was more effective than other examined groups. Strain Sz6 removed almost all colour in samples with triphenylmethane dyes regardless of dye concentration and structure and strain SDz3 only in samples with dyes concentration 0.05 gl^−^1. Higher content of dyes (0.1 gl^−^1) was removed by this strain in 62 % for crystal violet and 46 % for brilliant green.

Most researches are focused on the removal of individual dyes from wastewater, whereas the coloured wastewater contains a mixture of them. Relatively few studies on mixtures of dyes were conducted. In general, these studies were focused on dyes belonging to the same group (mainly azo group), (Saratale et al. 2010; Singh and Pakshirajan 2010; Tony et al. 2009; Vijaykumar et al. 2007). In addition, information about ecotoxicity of the biotransformation products is limited. In our study, we focused our attention on the removal efficiency of dyes mixture by two *Pseudomonas* strains. The mixture was prepared from dyes belonging to two different groups (azo and triphenylmethane) that was not investigated in any others studies. These two groups of dyes are more frequently used for dying processes and may be found in effluents. Such mixture may be more difficult for removal than single dyes as well as mixture of dyes from one family. Another goal of our study was to evaluate the ecotoxicological impact on aquatic organisms of biotransformation products.

## Materials and Methods

### Bacterial Strains Used in the Experiment

Bacterial strains used in experiment Sz6 and SDz3 were isolated from two different wastewater treatment plants in south Poland. Both selected strains were classified by the API 20NE test (Biomerieux) as *Pseudomonas fluorescens* (Zabłocka-Godlewska et al. 2012).

Species from *Pseudomonas* genus are Gram-negative, aerobic rod-shaped bacteria belonging to the family *Pseudomonadaceae*, almost all strains are motile (a single polar flagellum). As Gram-negative bacteria, they have thin cell wall containing up to 10 % of peptidoglycan and outer membrane composed of proteins, phospholipids and lipopolysaccharides. Strains *Pseudomonas fluorescens* are free-living bacteria, commonly found in environment (soil, water, effluents, surface of plants, animals, they are natural component of human and animal intestinal microflora), some of them may be pathogens (Salyers and Whitt 2000; Schlegel 1993).

### Influence of Dyes Concentration on Decolourisation Effectiveness

Influence of five different concentrations (0.01, 0.025, 0.05, 0.075 and 0.1 gl^−1^) of Evans blue (Zabłocka-Godlewska et al. 2012) and brilliant green (Table 1) on decolourisation effectiveness was estimated. The dyes mixture was prepared at the equal proportion (1:1) of both dyes. Influence of six different concentrations of dyes mixture (0.02, 0.04, 0.06, 0.08, 0.1 and 0.12 gl^−1^) on decolourisation effectiveness was estimated. Tube test on liquid Kimura medium was done in triplicate. Bacterial strains inoculums were prepared from 48 h slants (Nutrient Agar (Fluka Biochemika)) washed with physiological salt solution (suspension density estimated by the McFarland scale was 5). Filter-sterilised colourants were added to tubes containing 10 ml of 48-h old cultures in stationary growth phase (15 × 10^8^ cfu ml^−1^). After 6 days of incubation in 26 °C, absorbance was measured spectrophotometrically on UV–Vis Hitachi U-1900 (determined optimum wavelength for Evans blue was ${\mathtt{{\mathchar'26\mkern-9mu \lambda}}}_{\max }-606\kern0.5em \mathrm{nm}$, for brilliant green ${\mathtt{{\mathchar'26\mkern-9mu \lambda}}}_{\max }-624\kern0.5em \mathrm{nm}$ and dyes mixture ${\mathtt{{\mathchar'26\mkern-9mu \lambda}}}_{\max }-591\kern0.5em \mathrm{nm}$). Percentage of dye removal was calculated according to Formula 1.

**Table 1:**
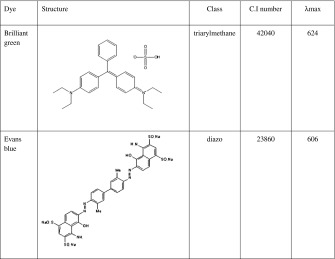
Characteristic of used dyes

1 $$ \mathrm{R}=\left(\left(C-S/C\right)\right.\times 100\% $$

R: Dye removal [%]
C: Concentration of dye in a control sample [mg l^−1^]
S: Residue concentration of dye in sample with bacteria biomass [mg l^−1^]

### Influence of Growth Conditions on Dyes Removal

The main experiment was prepared in a similar way as the previous test, the difference was that the inoculum (1 ml) was added to 300 ml flasks containing 100 ml of Kimura medium. On the basis of the results of previous tests, concentrations of dyes chosen for experiment with single dyes were 0.05 gl^−1^ for Evans blue (Zabłocka-Godlewska et al. 2012) and 0.1 gl^−1^ for brilliant green. Dyes concentration in sample with mixture was 0.08 gl^−1^ (0.04 gl^−1^ Evans blue and 0.04 gl^−1^ brilliant green (Sigma-Aldrich)). Water solution of dyes was filter-sterilised (Millipore cellulose filters Ø 0.20 μm) and added to 48-h old bacterial samples (stationary growth phase). Cultures were incubated in static, semistatic and shaken conditions in 26 °C. Dead biomass (biomass autoclaved for 20 min in 121 °C, 1.5 atm.) was used for estimation of biosorption. Absorbance was measured after 1, 6, 24, 48, 72, 96 and 120 h of incubation (UV–Vis spectrophotometer Hitachi U1900). The wavelengths for Evans blue (606 nm), brilliant green (624 nm) and dyes mixture (591 nm) were determined experimentally as the wave with maximal absorbance. Percentage of dye removal was calculated the same as previously according to Formula (1).

### Toxicity Evaluation

The zootoxicity was evaluated using *Daphnia magna* (OECD 202) and phytotoxicity using OECD *Lemna* sp. growth inhibition test no. 221. Tests were performed in quadruple. EC50 value was estimated. On the basis of these data, acute toxicity unit (TUa) was calculated (Formula 2) and toxicity class was established.

$$ \mathrm{TUa}=100/\mathrm{EC}50 $$

where

EC50 is the effective concentration of a wastewater sample that causes 50 % inhibition of tested organisms.

Samples were classified according to ACE 89/BE 2/D3 Final Report Commission EC (TUa < 0.4—non toxic (I class); 0.4 ≤ TUa < 1.0—low toxicity (II class); 1.0 ≤ TUa < 10—toxic (III class); 10 ≤ TUa ≤ 100—high toxicity (IV class); TUa > 100—extremely toxic (V class)).

## Results and Discussion

### Influence of Dyes Concentration on Decolourisation Effectiveness

**Fig. 1:**
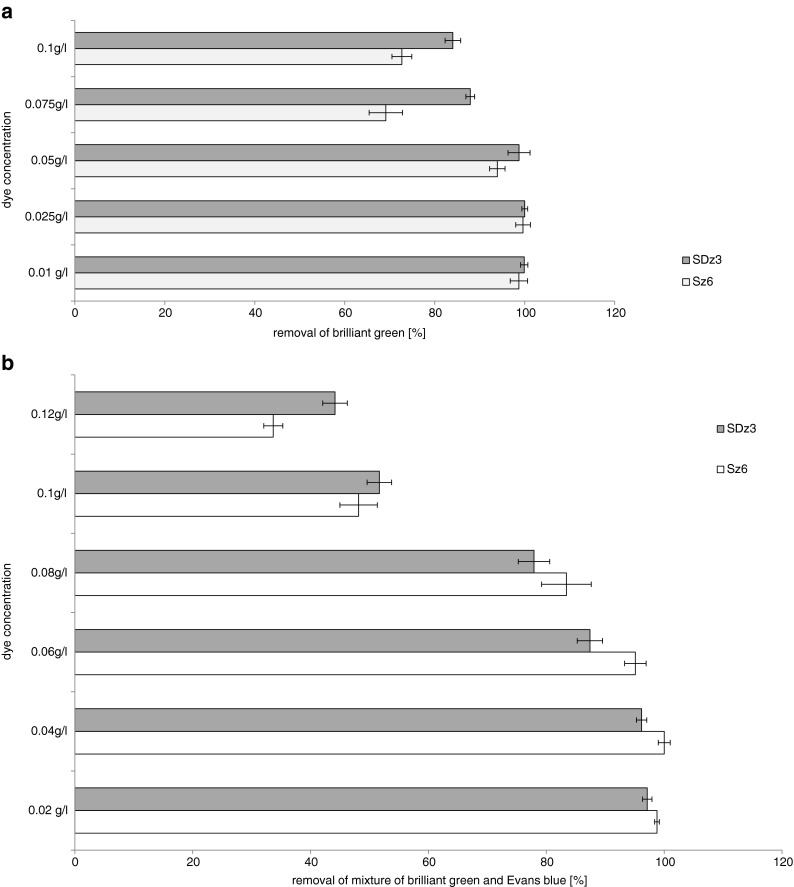
Influence of brilliant green (**a**) and dyes mixture (**b**) concentration on effectiveness of their removal

Influence of dyes concentrations (BG and mixture) was evaluated (Fig. 1a, b). Results for Evans blue were already presented (Zabłocka-Godlewska et al. 2012). Increase of Evans blue concentration up to 0.075gl−^1^ caused decrease of effectiveness of decolourisation by strain SDz3. For further study, the dye concentration 0.05gl−^1^ was chosen (Zabłocka-Godlewska et al. 2012). Brilliant green was better removed than Evans blue (Fig. 1a). Concentration 0.1gl−^1^ of brilliant green was removed by strain SDz3 in 84 % and by strain Sz6 in 72.7 %. Strain SDz3 almost completely removed this dye in concentrations 0.01–0.05 gl^−1^. Second tested strain (Sz6) removed BG in more than 95 % when concentration was between 0.01 and 0.05 gl^−1^. For further researches, this concentration of dye was chosen. Examined dyes belong to different groups, and different microbial decolourisation mechanism is involved in the process. The first step of microbial decolourisation of azo dyes is generally anaerobic process, which involves azoreductase enzymes activity and also oxygen insensitive azoredictase (Ola et al. 2010; Khalid et al. 2008; Nachiyar and Rajakumar 2005). Bacterial decolourisation of triphenylmethane dyes needs activity of triphenylmethane reductase (TMR) and in some cases cytochrome P450 (Wang et al. 2012). In our previous study (Zabłocka-Godlewska et al. 2012), strain Sz6 better removed brilliant green than Evans blue in concentration 0.1 gl^−1^ (96.5 and 84 %, respectively). In case of strain SDz3, significant differences were noticed in concentration 0.05 gl^−1^ (93.1 and 72.5 %, respectively). Also, in our other study (Przystaś et al. 2012), we observed that brilliant green was better removed than Evans blue when present in concentration 0.05 gl^−1^ in samples with different bacteria strains (regardless of incubation conditions). Differences between results reached for strains belonging to the same genus were also proved by Przystas et al. (2012). Two strains of *Pleurotus ostreatus* fungi had a different potential for the removal of the same dyes what may be connected with adaptation to the specific conditions in site of isolation. High impact of dye concentration on decolourisation effectiveness was also presented by Wu et al. (2009) and Solis et al. (2012).

Increase of concentration of dyes mixture had a significant influence on its removal by both strains (Fig. 1b). Concentrations between 0.02 and 0.08 gl^−1^ were removed by both tested strains in more than 75 %. Increase of dyes mixture concentration up to 0.1 gl^−1^ significantly decreased effectiveness of the process (removal below 50 %) As it is presented in Fig. 1b, the most appropriate mixture concentration for further studies was 0.08gl^−1^.

### Influence of Growth Conditions on Dyes Removal

**Fig. 2:**
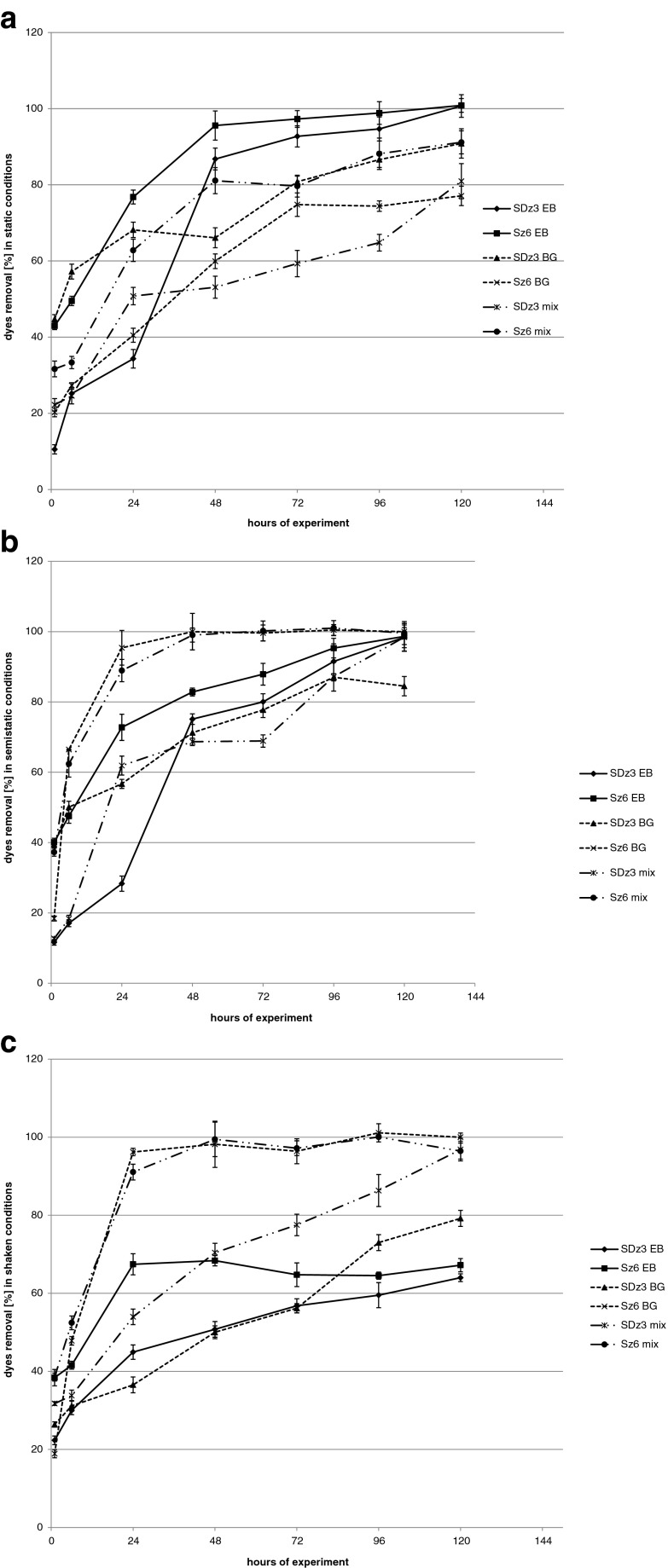
Percentage removal of dyes in static (**a**), semistatic (**b**) and shaken samples (**c**)

The best results of removal of Evans blue were reached in static samples (Fig. 2a). These results were presented in our previous publication (Zabłocka-Godlewska et al. 2012). Strain Sz6 was more effective in dye removal than SDz3 (after 48 h, about 96 and 87 % dye removal respectively). Finally, the dye was completely removed by both strains. In comparison with the results reached in samples with Evans blue, decolourisation of brilliant green in static conditions was lower (after 48 h SDz3 removed 66.15 % and Sz6 59.9 %). Initial concentration of brilliant green was two times higher than Evans blue. In spite of higher concentration removal of brilliant green by strain SDz3 at the end of experiment reached 91.16 %.

Initially, in semistatic conditions, results of removal of Evans blue (Fig. 2b) were not so good as in static samples (after 48 h, SDz3 removed 75.1 % and Sz6 82.8 %). After 120 h of experiment, decolourisation by Sz6 strain reached 100 % as well as dyes mixture and brilliant green. In comparison with static samples in semistatic samples, acceleration of brilliant green and dyes mixture removal by strain Sz6 was observed. After 24 h of experiment, the level of removal was higher than 90 % and after 48 h, complete decolourisation was observed. Effectiveness of brilliant green removal by strain SDz3 was comparable in static and semistatic conditions (after 120 h, 91.2 and 84.5 %, respectively). Presented results for brilliant green are similar with our previous results (Przystaś et al. 2012). Regardless of used strain, the best results of BG decolourisation were reached in shaken conditions what can suggest that oxygen is important in this process. In opposite to the result presented above for *Pseudomonas fluorescens* strains (Sz6 and SDz3), Evans blue was better removed by *Chryseomonas luteola* and *Burkholderia cepacia* tested earlier (Przystaś et al. 2012) in shaken samples. All of these strains are aerobic, so these results suggest involving of different biodegradation mechanisms. Higher effectiveness of triphenylmethane crystal violet decolourisation in shaken than static conditions was also reached for *Citrobacter* sp. by An et al. (2002).

In present studies, a positive influence of agitation on triphenylmethane dye removal by both *Pseudomonas* strains was noticed. In opposite to our results, Wang et al. (2011) stated that in case of *Achromobacter xylosoxidans*, MG1 sample agitation had no influence on triphenylmetane decolourisation. It suggests contribution of different decolourisation pathways in process. Current knowledge about triphenylmethane dyes decolourisation mechanisms is still in its infancy (Wang et al. 2011, 2012). In study of biodegradation of triphenylmethane malachite green (MG) presented by Jang et al. (2005), the triphenylomethane reductase (TMR) was shown to be responsible for the conversion of MG to colourless leucomalachite green (LMG). Wang et al. (2012) speculate that MG degradation by *Exiguobacterium* sp. involves the reaction of *N*-demethylation, reduction, benzene ring removal and oxidation. In the postulated pathway, the first step transforming MG to LMG is through the enzyme TMR. Cytochrome P450 most likely participated in further transformation of MG. This superfamily of hemoproteins has been also reported to be involved in triphenylomethane dyes degradation by *Mycobacteria* (Jones and Falkinham 2003; Wang et al. 2012). Wang et al. (2011) highlighted that decolourisation mechanism of these type of dyes might be both the intracellular and extracellular. Differences in rate and abilities for dyes decolourisation between strains are probably connected with various mechanisms of dye transformation as it was presented by Wang et al. (2012) and Wu et al. (2012).

Samples agitation had a negative influence on the effectiveness of Evans blue decolourisation (Fig. 2c). Results are also presented and described in the previous publication (Zabłocka-Godlewska et al. 2012). After 120 h, strain SDz3 removed 64.0 % and Sz6 67.2 % of dye. Decolourisation of azo dyes may occur under strictly anaerobic, facultative anaerobic or aerobic conditions. It depends on the strain and mechanism of biotransformation. Agitation caused increase of oxygen concentration in samples. Effect of agitation and changes of oxygen concentration on azoreductase activity is widely described in literature (Saratale et al. 2011). Worse decolourisation results in semistatic and shaken samples point out on possible oxygen inhibition effect on azoreductases activity. Azoreductases are responsible for the first step on decolourisation of azo dyes into simpler aromatic compounds (Saratale et al. 2011).

Results of dyes mixture removal were similar in shaken and semistatic samples (100 % dye removal after 48 h) for strain Sz6 (Fig. 2b). For this strain, results are similar to these reached for brilliant green. The rate of dyes mixture removal by strain SDz3 was slower (70.4 % in shaken sample and 68.7 % in semistatic samples after 48 h). After 96 h of experiment, difference between results reached for both strains in semistatic and shaken conditions was lower than 15 % and finally (after 120 h) strain SDz3 completely removed dyes mixture (Fig. 2b,c). In static conditions, final removal of mixture was lower, 91.2 % for strain Sz6 and 81 % for SDz3. These results were similar to the brilliant green removal in static sample by both strains and were lower than Evans blue decolourisation effect (Fig. 2a). Decolourisation of mixture, as well as individual tested dyes was faster in case of strain Sz6.

High decolourisation potential of *Pseudomonas* strains for dyes decolourisation is presented in literature. Chen (2002) used *Pseudomonas luteola* for decolourisation of reactive azo dyes. He proved that this process is connected with azoreductase enzyme activity and oxygen sensitive. Silveira et al. (2009) used in the experiment three different *Pseudomonas* species for decolourisation of 14 industrial textile dyes. The experiment was done under static anoxic conditions and proved high decolourisation potential of *Pseudomonas aeruginosa* and *Pseudomonas oleovorans*. Another study with *Pseudomonas* strains was carried by Wu et al. (2009). Decolourisation of azo and tripenymethane (TPM) dyes was examined. Crystal violet, malachite green and brilliant green (TPM) were almost completely removed by *Pseudomonas otitidis* WL-13 up to concentration 100 μmoll^−1^, where methyl red and Congo red (azo dyes) were removed in less than 45 % when were present in sample in concentration 10 μmoll^−1^ (12 h experiment). Jadhav et al. (2010) studied the bacterial consortium composed from three *Pseudomonas* species in the experiment with azo dyes. Consortium had better decolourisation results than pure cultures. One of the strains (*Pseudomonas* sp. SUK1) used in studies describe above was also examined by Kalyani et al. (2009). The experiment was done with azo Reactive Red 2 in static conditions. Tested *Pseudomonas* strain was capable utilise toxic RR2 in concentration 5gl^−1^ in more than 70 % after 108 h and more than 90 % in concentration 2gl^−1^during 48 h. This strain decolorized with a great results azo dyes mixture. Decolourisation abilities of *Pseudomonas aeruginosa* BCH was examined by Jadhav et al. (2011). Monochloro-sulphonated azo dye Remazol Red was degraded up to 97 % within 20 min. in static conditions.

**Fig. 3:**
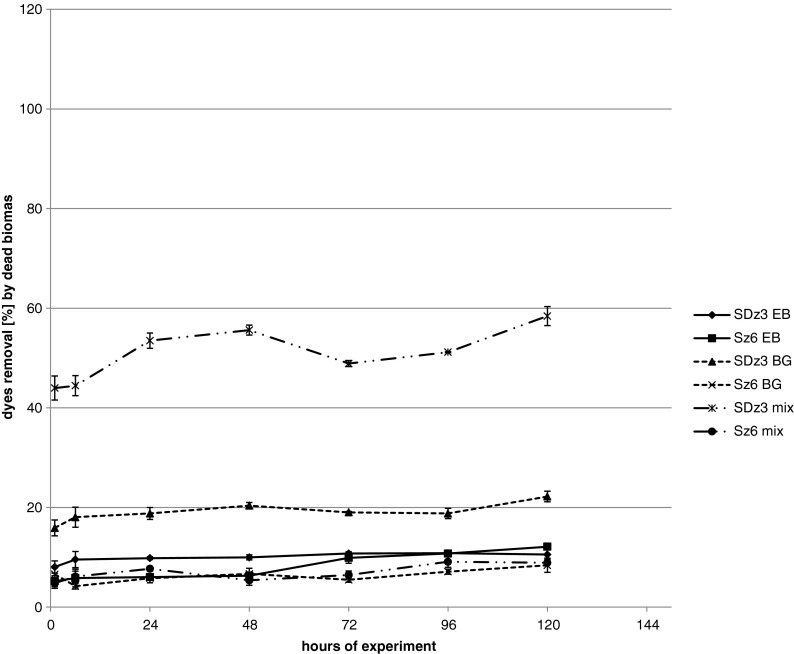
Removal of dyes in samples with dead biomass

**Fig. 4:**
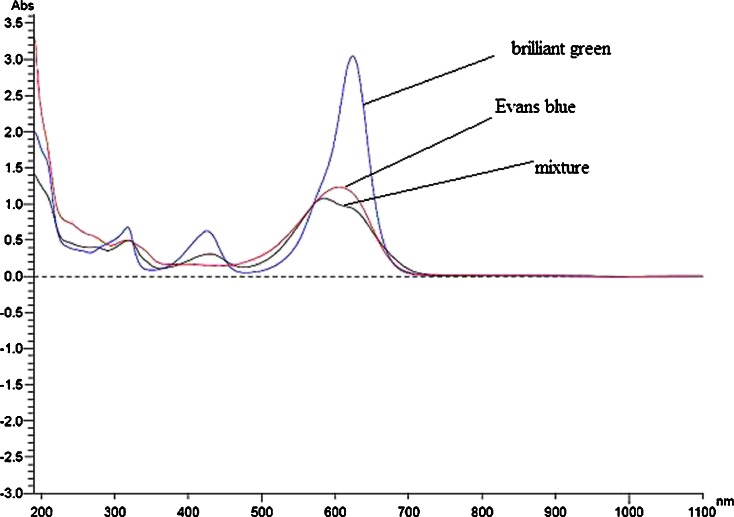
UV-Visible spectral scans

Dead biomass was used for evaluation of sorption (Fig. 3). Sorption of dyes mixture by Sz6 was approximately 9 %. The best results of dye sorption were noticed in samples with dyes mixture with strain SDz3 (from 44 % after 1 h up to 58.4 % dye removal after 120 h). As it is visible on Fig. 4, interaction between azo Evans blue and triphenylmethane brilliant green in mixture was observed. UV-Visible spectral scans (Fig. 4) show the displacement of major peak to 591 nm. Probably, this phenomenon caused higher sorption of dyes mixture by strain SDz3. These results were surprising because so high sorption was not observed in sample with individual dyes. For strain SDz3, sorption of brilliant green after 120 h was approximately 20 %, Evans blue approximately 10 % and for stain Sz6 approximately 8 and 12 %, respectively. Explanation of this phenomenon observed for dye mixture is difficult. Maybe it is connected with differences in quality and quantity of exopolysaccharides exuded by both tested strains what may influence on dyes sorption abilities when interaction between dyes happens. Wu et al. (2009) reported high effectiveness of dye removal by *Pseudomonas otitidis* WL-13 connected with adsorption on the biomass. Strain removed 95 % of brilliant green and malachite green in concentration 500 μmol l^−1^ after 12 h.

### Toxicity Evaluation

Zootoxicity as well as phytotoxicity (Table 2) were also evaluated. Generally, zootoxicity and phytotoxicity after decolourisation process were lower or at the same level as in controls sample with dyes. Only in case of strain Sz6 with Evans blue incubated in static conditions zootoxicity increased from IV to V class. In these samples, complete decolourisation was observed and as it was suggested above transformation of azo dyes by azoreductases to toxic aromatic amines. In spite of high effectiveness of Evans blue removal in semistatic and shaken conditions, zootoxicity was at the same level as in control sample. In sample with dead biomass, removal reached less than 15 % but the zootoxicity decreased to III class. Such results of experiment proved that toxic metabolites were produced during Evans blue biotransformation by strain Sz6. In opposite to Sz6 in samples with SDz3, significant decrease of zootoxicity was observed (from IV to III class).

**Table 2:** Results of toxicity tests

Strain	Culture conditions	Dye	*Daphnia magna*			*Lemna minor*		
			EC_50_	TUa	Toxicity class	EC_50_	TUa	Toxicity class
Sz6	Static	EB^b^	0.8 ± 0.32	125	V	n.d.^a^	<0.4	I
		BG	1.56 ± 0.15	64.1	IV	7.23 ± 0.12	13.8	IV
		Mix	4.69 ± 0.09	21.3	IV	7.81 ± 0.09	12.8	IV
	Semistatic	EB^b^	9.43 ± 1.08	10.6	IV	n.d.^a^	<0.4	I
		BG	18.69 ± 2.17	5.35	III	8 ± 1.8	12.5	IV
		Mix	4.42 ± 0.27	22.6	IV	9.43 ± 0.79	10.6	IV
	Shaken	EB^b^	4.69 ± 1.70	21.3	IV	20.00 ± 4.10	5	III
		BG	4.69 ± 0.46	21.3	IV	10.42 ± 1.1	9.6	III
		Mix	2.31 ± 0.1	43.3	IV	9.43 ± 1.44	10.6	IV
	Dead biomass	EB^b^	11.36 ± 0.96	8.8	III	23.26 ± 8.20	4.3	III
		BG	0.8 ± 0.27	125	V	166.67 ± 7.23	0.6	II
		Mix	1.56 ± 0.16	64.1	IV	4.69 ± 0.02	21.3	IV
SDz3	Static	EB^b^	12.5 ± 2.50	8.0	III	n.d.^a^	<0.4	I
		BG	18.69 ± 3.1	5.35	III	111.11 ± 7.51	0.9	II
		Mix	47.62 ± 3.7	2.1	III	1.56 ± 0.47	64.1	IV
	Semistatic	EB^b^	37.04 ± 2.80	2.7	III	n.d.^a^	<0.4	I
		BG	18.69 ± 0.17	5.35	III	111.11 ± 5.66	0.9	II
		Mix	11.49 ± 2.2	8.7	III	1.56 ± 0.23	64.1	IV
	Shaken	EB^b^	18.87 ± 3.20	5.3	III	76.92 ± 8.30	1.3	III
		BG	9.43 ± 0.76	10.6	IV	35.71 ± 2.13	2.8	III
		Mix	7.81 ± 0.16	12.8	IV	1.56 ± 0.11	64.1	IV
	Dead biomass	EB^b^	10.64 ± 1.80	9.4	III	24.39 ± 9.50	4.1	III
		BG	1.76 ± 0.12	56.7	IV	3.07 ± 0.67	32.6	IV
		Mix	52.63 ± 5.14	1.9	III	1.56 ± 0.22	64.1	IV
Controls		EB^b^	9.43 ± 0.22	10.6	IV	22.22 ± 2.10	4.5	III
		BG	0.98 ± 0.07	102	V	3.07 ± 0.52	32.6	IV
		Mix	0.92 ± 0.15	108.7	V	1.2 ± 0.01	83.3	IV

^a^Not detected

^b^Results of toxicity of Evans blue decolourization end-products were published in Zabłocka-Godlewska et al. 2012

Franciscon et al. (2009a) reported that under microaerophilic conditions, toxicity of metabolites of azo dyes transformed by *Staphylococcus arlettae* for *Daphnia magna* decreased and in aerated samples, no toxicity was detected. The same results were reached by Franciscon et al. (2009b) for *Klebsiella* sp. strain VN-31.

High effectiveness of brilliant green removal by living biomass (>77 %) was connected with significant decrease of zootoxicity (from V to IV or III class). Third class of zootoxicity was estimated in static and semistatic samples with SDz3 and semistatic with Sz6. Slight adsorption of brilliant green by dead biomass of SDz3 resulted in decrease of zootoxicity to III class when samples with dead biomass of Sz6 were extremely toxic (V class). Our previous study (Przystas et al. 2012) showed that bacterial decolourisation of brilliant green by *Chryseomonas luteola* and *Burkholderia cepacia* leads to decrease of zootoxicity but phytotoxicity was at the same level.

Dyes mixture was also effectively removed by living biomass of tested strains, and in all samples after 120 h of experiment, decrease of zootoxicity was observed (from V to IV class in case of strain Sz6, regardless of incubation method). High effectiveness of colour removal by strain SDz3 in semistatic and static conditions as well as by dead biomass caused decrease of zootoxicity to III class. Shaking connected with oxygenation of samples might change the metabolic pathway of dyes transformation that is why no so significant decrease was noticed. Even if decolourisation is very high, metabolites produced during the process might be different.

*Lemna minor* was more resistant to dyes as well as to bacterial metabolites. High removal of dyes mixture had no influence on phytotoxicity. Effective removal of brilliant green was connected with the decrease of phytotoxicity in samples with shaken biomass of strains Sz6 as well as strain SDz3 (III class), static samples with the same strain (II class) and dead biomass of strain Sz6 (even to II class of toxicity). Samples with Evans blue were classified even to I class of phytotoxicity (Sz6 and SDz3 incubated in static and semistatic conditions). Other samples (including dead biomass of both strains) were classified the same as controls to III class of phytotoxicity.

Findings of Cui et al. (2012) suggest that the biodegradation products of azo dyes transformation by bacterial consortia are less toxic to plants (*Brassica pekinensis*) than tested dyes. It proves that bacterial decolourisation of azo dyes can lead to their detoxification. Sheth and Dave (2009) reported that metabolites of Reactive Red biodegradation by *Pseudomonas aeruginosa* NGKCTS did not inhibit growth of soil microorganisms. Jadhav et al. (2010) reported no changes in DNA damage after decolourisation of mixture of reactive azo dyes by *Pseudomonas* consortium and weaker inhibition of seeds germination in samples with dyes metabolites. Decolourisation of Evans blue and brilliant green mixture by fungal strains was evaluated by Przystaś et al. (2013). Decrease of zootoxicity after treatment was noticed for all tested modifications and strains. In case of phytotoxicity, decrease was observed only in samples treated by single strains.

## Conclusions

The present study of dyes decolourisation confirms high decolourisation effectiveness of Evans blue (EB), brilliant green (BG) and mixture (Mix) of them by two *Pseudomonas* strains (Sz6 and SDz3) isolated from different sites. Incubation conditions affected the rate of decolourisation. The best results were reached in shaken and semistatic samples. After 24 h of experiment in semistatic samples, BG removal reached up to 95.4 %, EB 72.8 % and dyes mixture 88.9 %. After 120 h, all tested dyes were completely removed. In most cases, strain Sz6 removed dyes faster and better than SDz3. Decrease of phyto- and zootoxicity of samples after 120 h was observed. Such results confirm possible usage of isolated strains to effective, cheap and environmental safe dyes removal also if they are present in wastewater in mixture.
